# The evolution of China's youth sport policies—a systematic analysis based on national policy texts 2013–2023

**DOI:** 10.3389/fspor.2026.1854324

**Published:** 2026-06-05

**Authors:** Pengfei Shi, Alan Bairner

**Affiliations:** 1 School of Management, Beijing Sport University, Beijing, China; 2 School of Sport, Exercise and Health Sciences, Loughborough University, Loughborough, United Kingdom

**Keywords:** Five-Year Plan, health promotion, incrementalism, policy evolution, sports-education integration, youth sports

## Abstract

Youth sports constitute a significant global policy concern, with governments commonly employing policy interventions to guide their development. Understanding how national priorities within youth sport evolve over time is essential for appreciating the broader trajectory of youth development governance. Drawing on incrementalism theory, this study employs bibliometric analysis, content analysis, and social network analysis to examine the evolution of policy priorities across 105 youth sports policies issued by the Chinese central government between 2013 and 2023. The findings indicate that the focus of China's youth sports policies has undergone a phased evolution: from cultivating competitive sports talents (18th NCCPC period 2012–2016), to integrating sports with education (19th NCCPC period 2017–2021), and subsequently to establishing a health-first principle (20th NCCPC period 2022–present). This progression closely aligns with the country's major political cycles and Five-Year Plans. The study further reveals that, how seemingly marginal early interventions can serve as institutional anchors for subsequent reform, while also identifying coordination challenges between the sports and education systems due to policy ambiguity. The conclusions drawn here may therefore be most applicable to contexts characterised by strong central coordination capacity and developmental orientations toward youth wellbeing.

## Introduction

Youth sport occupies a central position in the global sport policy landscape. Across diverse national contexts, governments have increasingly recognised its dual function: fostering the physical health, character development, and lifelong active habits of young people on the one hand, and cultivating elite athletic reserve talent on the other (1–3). These policies are distinguished from broader sport policy by their age-specific orientation, encompassing physical education, youth participation promotion, public health improvement, and high-performance talent pipelines (4–6). As national circumstances evolve, the relative emphasis placed on these functions within youth sport policy is subject to continuous adjustment, shaped by political priorities, socio-economic conditions, and emerging public health challenges (7). Tracing how such policy priorities shift over time is essential for understanding the broader trajectory of youth sport governance. The present study examines this process of priority evolution within the context of China's youth sport policies from 2013 to 2023.

As a country with a strong central government, China relies on policy formulation and implementation as one of the primary means of achieving social development governance (8, 9). In recent years, Chinese athletes have performed well in various international sports events, with numerous medal winners originating from youth groups. This success stems not only from China's large youth population providing a crucial foundation for youth sports development but also from China's youth sports policies and the Chinese government's prioritisation of youth sports development (10). Within China's whole-of-nation strategy (*JU GUO TI ZHI*), a core function of youth sport lies in cultivating reserve talent for competitive sport. Many young people are selected from an early age to enter specialised, centralised training systems to enhance athletic skills and pursue excellence in competitions. However, this historically dominant focus on competitive sporting achievements has, to some extent, led to the relative neglect of youth sport's health promotion function. The youth population faces persistent challenges to physical and mental wellbeing, including insufficient physical activity, rising obesity rates, and a sharp increase in myopia prevalence (11, 12).

In response to these challenges, the Chinese government has since 2013 launched a series of targeted policies to cultivate physical fitness, moral development, and lifelong exercise habits. These policies, spanning the periods of three National Congresses of the Communist Party of China (18th, 19th, and 20th NCCPC) and three Five-Year Plans (12th–14th), have progressively expanded the scope of youth sport policy to encompass health promotion, sports-education integration, and holistic youth development. Typical policies include the *13th Five-Year Plan for Youth Sports (2016)* (88) and *Youth Sports Activity Promotion Plan (2017)* (89). These policies seek to systematically improve the youth sports development system through organisational optimisation, venue infrastructure development, the provision of public services, and the integration of sports within schools, thereby creating favourable conditions and robust support for youth participation in sporting activities.

A critical driver of this policy evolution has been the escalating concern over adolescent physical and mental health. Policies such as the *Opinions on Further Reducing the Homework and Off-Campus Training Burden of Students in Compulsory Education (2021)*, commonly known as the “*Double Reduction” policy*, can be understood as indirect evidence of the severity of health challenges facing Chinese youth. The excessive academic burden and school pressure implied by such policies have contributed to rising rates of myopia, obesity, and sedentary behaviour among adolescents. In this context, sport has been increasingly positioned as a vital instrument for promoting youth health, and health-related content within youth sport policies has expanded correspondingly across the study period.

The cumulative impact of these policy developments is evident in both participation growth and health indicator improvements. According to the 2020 Survey Bulletin on the Status of National Fitness Activities issued by China's National Centre for National Physical Fitness Monitoring (13), in 2014, 62.6% of children and adolescents (ages 7–18) engaged in physical exercise at least once a week, rising to 81.1% by 2020. Similarly, the percentage of youth frequently participating in sports grew from 50.2% in 2014 to 55.9% in 2020. These improvements reflect the growing effectiveness of youth sport policies in promoting physical participation and fostering healthier lifestyles among the adolescent population.

It is therefore evident that China's youth sport policies have undergone significant evolution over the past decade, with a discernible shift in developmental priorities. This study aims to trace and analyse this evolution systematically, examining how the content and focus of youth sport policies have changed across political cycles, and how health-related priorities have gained increasing prominence within the policy framework.

## Literature review

In recent years, the field of sports policy research has produced substantial advancements globally. Studies on youth sports policies remain predominantly concentrated in Europe and North America, with scholars conducting extensive research and discussion on two key aspects: the development of youth athletes and the promotion of youth health and wellbeing. Research perspectives cover comparative frameworks, historical evolution, and implementation challenges of youth sports policies across countries. On the one hand, scholars have focused intensely on youth athlete development, including pathways for elite athlete cultivation, influencing factors, and policy execution. Kårhus (6) examined the integration of elite sport as legitimate educational knowledge within Norway's national curriculum, analysing the discourses underpinning this shift and its implications for aspiring athletes at secondary school level. Kristiansen et al. (14) conducted a cross-national comparative analysis of elite youth sport policies and governance, exploring global trends in youth athlete development, stakeholder attitudes (including governments and national governing bodies), and the balance between talent selection and athlete welfare across multiple nations.

On the other hand, youth sport's contribution to adolescent health and wellbeing is equally significant. Green and Collins (5) employed path dependency theory to compare Australia and Finland's sport development policies, revealing differences between Australia's elite-focused approach and Finland's “sport for all” philosophy. Their analysis illustrated how historical political decisions shaped the subsequent trajectory of youth sport priorities, demonstrating the path-dependent nature of policy evolution. Hernández and Pardo (15) compared school sports objectives in Spain and France through organisational perspectives and stakeholder interviews, examining how educational goals such as physical literacy and social integration are achieved under different national systems. Their findings highlighted the significant role of institutional arrangements in shaping the balance between health-oriented and performance-oriented youth sport objectives.

Complementing this, Lindsey et al. (16) conducted an expansive scoping review and synthesis of youth sport policy research spanning the two decades from 2000 to 2020. While examining core issues such as the formulation, content, and effectiveness of youth sport policies, they highlight research gaps in theoretical and methodological approaches within this field. They further stress the critical necessity to expand youth sport policy research beyond Western contexts to address global disparities in youth participation, athlete development, and resource utilisation, thus underscoring the critical importance of increasing research evidence from China, a nation within the Asia-Pacific region possessing a substantial youth population base.

In the context of China's rapidly evolving youth sports policies, related research has progressively deepened, particularly in examining the macro-level of the national sports policy framework. It is evident, however, that competitive sports, with elite athletes as their primary focus, remain central to China's sports policy studies. For instance, this is evident in how youth sports policy implementation aims to enhance competitive sports standards among young people. Tan et al. (17) examined policy transfer phenomena in elite sports development using China's elite swimming programme as a case study, revealing how international experiences have been adapted and localised since the early 21st century to optimise organisational structures and enhance competitive outcomes. Wang et al. (18) focused on the sustainable development of provincial-level competitive sports in China, analysing and evaluating models for resource allocation, infrastructure, and long-term sustainability to support the national goal of a leading sporting nation. Peng et al. (19) examined the implementation of China's 2015 youth football reform, revealing policy contradictions stemming from organisational barriers, resource constraints, and stakeholder conflicts. They assessed how these obstacles hindered the establishment of a unified youth training system designed to simultaneously elevate grassroots and elite football standards. Further research addresses talent selection for competitive sports and participation promotion within youth sports. Chen and Chen (2) conduct a detailed analysis of China's youth sports, emphasising the central government's pivotal role in determining development patterns through national policy initiatives. This is particularly evident in areas such as physical education curricula, school sports programmes, and efforts to promote universal youth participation and talent identification. to a certain extent, this promotion of youth sports participation also serves to enrich the talent pool for competitive sports.

Notwithstanding these contributions, a notable gap persists in the existing literature. Whilst individual studies have examined specific policy episodes or institutional mechanisms, few have undertaken a systematic, longitudinal analysis of how the substantive priorities within national youth sport policy texts evolve across political cycles. In particular, limited attention has been paid to tracing the incremental expansion of health-related content within youth sport policy over an extended period, or to examining how this evolution intersects with China's distinctive governance rhythms, including the Five-Year Plan cycles and Party Congress agendas. This represents a significant omission, given that China's centralised policy-making system and its periodic strategic planning mechanisms offer a distinctive context for understanding how youth sport priorities are shaped, adjusted, and rebalanced over time. Against this backdrop, this study presents a case study from the Asia-Pacific region. Through a decadal textual analysis, it examines the incremental, cycle-anchored characteristics of the evolution of youth sports policies and tests existing theoretical framework (such as Lindblom's incrementalism) in a non-Western context, which aligns with the agenda proposed by Lindsey et al. (16).

### Theoretical framework

Given that the present study seeks to investigate the staged evolution of policy priorities for youth sport in China between 2013 and 2023, an incrementalist perspective is adopted as the analytical framework (20–22). In contrast to the Multiple Streams Framework (MSF), which foregrounds the role of focusing events in triggering policy change (23, 24), and to Punctuated Equilibrium Theory (PET), which casts policy trajectories as long stretches of stasis interrupted by abrupt punctuations (25, 26), incrementalism holds that policy change typically proceeds through a series of gradual, marginal adjustments rather than through a wholesale overturning of existing arrangements. Its account of small-scale, step-by-step policy adjustment offers a more accurate fit with the patterns observed across the past decade of youth sport policy in China. Within this framework, decision-makers are conceived of as boundedly rational actors who, unable to canvass all possibilities from abstract goals, instead take the status quo as their reference point and engage in successive limited comparisons across a small set of alternatives that diverge only marginally from existing arrangements; satisfactory rather than optimal policy outcomes are accumulated through iterative marginal adjustments, repeated trial and error, and partisan mutual adjustment among multiple actors (20, 27). Hayes (22) further argues that, precisely because of decision-makers’ cognitive limits, the costs of information acquisition and the persistence of value disagreements, substantive change in public policy is almost invariably achieved through such seriality rather than through one-off reconstruction.

Our study draws principally on two core assumptions of the incrementalist perspective (28): 1. human cognition and rationality are inherently bounded, such that decision-makers cannot exhaustively compute all policy consequences; and 2. valid policy knowledge originates in practice, accumulates through implementation and is refined via feedback.

In concrete terms, the multi-objective complexity confronting youth sport in China is such that no single policy can deliver an optimal configuration across all of its objectives at once. In the practical realities of governing youth sport development in China, the government's response to emerging problems—through localised revisions of existing policy frameworks and the gradual embedding of new instruments—is precisely the realistic course of action available under bounded rationality. The policy tradition of “piloting first, scaling up later”, together with its institutionalised cadence, in turn embodies the integration of practical reasoning with applied knowledge. In their analysis of China's Five-Year Plan (FYP) system, Heilmann and Melton (29) characterise the system not as a top-down unitary blueprint but as a planning coordination and evaluation cycle, with each planning period embedding institutionalised junctures of review, revision and redeployment that lend policy adjustment a regular cadence. Together with the regularly convened National Congresses of the Chinese Communist Party (CCP), this rhythm furnishes a systematic institutional channel through which the government can incrementally revise policy content and accumulate applied knowledge in light of prior implementation feedback. The 2013–2023 period covered by the present study spans the latter portion of the 12th FYP, the entirety of the 13th FYP and the early phase of the 14th FYP, as well as the 18th, 19th and 20th National Congresses of the CCP, providing an ideal temporal window for observing precisely this form of incremental policy evolution, anchored in practical feedback and structured by institutional cadence.

### Research questions

Building upon the foregoing review, this study addresses the following core questions: (1) How have the substantive priorities within China's national youth sport policies evolved across the periods of the 18th, 19th, and 20th National Congresses of the CPC (2013–2023)? (2) What are the phased characteristics and influencing factors of this policy evolution, and how do they correspond to China's political cycles and Five-Year Plans? (3) What roles do the core government departments (GAS, MOE, NHC) play in shaping youth sport policy priorities, and how do their collaborative patterns evolve across the study period?

The significance of this research lies in three aspects. First, it provides a systematic, longitudinal analysis of youth sport policy evolution based on continuous policy text data, moving beyond fragmented case-based narratives. Second, it integrates bibliometric analysis, content analysis, and social network analysis to quantify policy shifts, examine key policy content, and assess the roles played by various government departments. Third, it focuses on the critical decade from 2013 to 2023, spanning three Party Congresses and three Five-Year Plans, providing a vital historical snapshot for understanding how youth sport priorities are incrementally rebalanced within China's centralised governance system.

## Research methods

Most existing sports policy research is grounded in social constructivism and tends to use qualitative research methods such as interviews, literature analysis, case studies, and logical reasoning to delve into the underlying values and implementation logic behind policy texts (7, 30). It is worth noting that the application of quantitative research methods in the field of sports policy (including youth sports policy research) remains relatively limited (16). However, in other public policy fields, quantitative analysis has been widely applied, such as text mining-based policy tool strength measurement (31), quantitative assessment of policy performance (32), policy network structure analysis and the construction of multi-dimensional policy indicator models (18), demonstrating the feasibility and distinctive contributions of quantitative methods in uncovering policy impacts and tracing evolutionary trajectories.

This study collected Chinese youth sports policy documents to conduct bibliometric analysis, content analysis, and social network analysis. This combination of methods allowed for the systematic processing of the policy texts issued over a decade. By analysing annual policy issuance, policy authority hierarchies, and other factors, it objectively reveals cyclical patterns in policy release and the evolution of thematic hotspots (33). Moreover, by examining co-issuing institutions, this study constructed inter-departmental collaboration networks. These visualise the interaction intensity and structural relationships between core departments, such as the General Administration of Sport and the Ministry of Education, during policy formulation. This approach illuminated the structural causes underlying either fragmented governance or coordinated efficiency (34). As a result, it provided insights into the implementation logic underlying policy texts and the shifting priorities in national youth sports development across different phases, enabling a comprehensive portrayal of the evolution of China's youth sport policy priorities over the study period.

### Data collection

This study collected youth sports policies promulgated by China's central government agencies (mainly the State Council, General Administration of Sport, Ministry of Education, and National Health Commission) between 2013 and 2023. This decade captures pivotal institutional reforms, complete implementation cycles of key national initiatives, and critical developmental stages of the youth sport. Within China's centralised governance system, central-level policies establish core objectives and jurisdictional parameters for youth sports, with provincial adaptations deriving from these institutional blueprints (35, 36). The period aligns with three quinquennial National Congresses of the Communist Party of China (18th/19th/20th NCCPC) and three Five-Year Plans (12th–14th), wherein the NCCPC (as the supreme agenda-setting forum) endowed policy frameworks with strategic coherence (37). This ten-year cycle witnessed youth sports’ ascendance from administrative routine to national priority, culminating in the 2022 Sports Law revision that mandated national youth sports promotion.

Policy texts were systematically retrieved from four authoritative sources: the Central People's Government portal, State Council databases, and Peking University's *Magic Weapon* legal repository (http://www.pkulaw.com). Boolean searches for *youth sports, school sports,* and *physical education* (from 1 January 2013 to 31 December 2023) yielded 347 initial documents. Application of five inclusion criteria refined the corpus to 105 definitive policies: (1) State Council/ministerial issuance; (2) 2013–2023 publication; (3) binding/guidance document types (laws, regulations, action plans); (4) substantive youth sports focus; (5) operational validity during the study period.

### Data analysis

This study first quantified the annual distribution, types, and release patterns of policies through bibliometric analysis. Next, the study identified the priorities, developmental characteristics, and objectives of China's youth sports policies through content analysis. Finally, social network analysis (SNA) was employed to examine collaborative patterns among key government departments involved in policy issuance. Social network analysis constitutes a predominantly quantitative research methodology (38), suitable for exploring patterns of inter-organisational collaboration (39, 40). Network visualisations were generated using Gephi 0.10.1 software (41, 42).

For the bibliometric component, four indicators were computed directly from the metadata of the 105-document policy corpus: (i) annual issuance frequency (the total number of policies released per calendar year, used to detect cyclical surges); (ii) cumulative issuance (the running total, used to gauge the overall expansion of the policy stock); (iii) the distribution of issuing bodies (disaggregated by the State Council, the General Administration of Sport, the Ministry of Education, the National Health Commission and other ministries, used to trace which actors have shouldered the policy-making remit); and (iv) the hierarchical level of policy instruments (the four-tier legal hierarchy described in the “Authority Level” sub-section, used to gauge the binding force of each text). All counts were generated in Microsoft Excel 2021 and manually cross-checked against the original PDF and HTML sources in order to eliminate duplicates and superseded versions.

For the social network analysis, in addition to descriptive visualisation, four standard network- and node-level metrics were computed in Gephi 0.10.1 to permit more rigorous structural comparison: (a) network density (the ratio of observed ties to all possible ties, reflecting the overall integration of the co-issuance network); (b) weighted degree centrality (the sum of each ministry's co-issuance frequencies, used to identify dominant policy actors); (c) betweenness centrality (the extent to which a given ministry lies on the shortest paths between other actors, indicating its bridging role); and (d) modularity Q (43), computed via the Louvain algorithm at a resolution of 1.0 to detect densely connected sub-communities. These metrics were calculated for the full decade as well as for each of the three sub-periods, enabling the structural evolution of the cooperation network to be traced across phases.

To trace the evolution of policy priorities and focus, this study employed NVivo 14 to systematically code 105 youth sport policy documents promulgated between 2013 and 2023, thereby constructing a multidimensional coding framework (Table 1). The framework is organised around three primary codes: Primary Code (1) targets specific cohorts of athletically gifted young people through elite selection and high-performance training systems; Primary Code (2) addresses the broader youth population through community- and population-level health interventions; and Primary Code (3) takes school-enrolled pupils as the principal beneficiary group, operating through the incorporation of physical education into the formal curriculum and the establishment of institutional arrangements that link sport with schooling. To further illuminate the deployment of policy instruments within the governance architecture through which these priorities are implemented, the study drew upon the NATO typology proposed by Hood and Margetts (44) to characterise the policy instruments and modes of implementation associated with each secondary code. The instrument types comprise nodality (information-based tools), authority (legal and regulatory tools), treasure (fiscal tools) and organisation (direct-delivery tools).

**Table 1:** Coding framework of youth sports policy documents in China.

Primary Codes	Secondary Codes	Meanings of Secondary Codes	Policy Instruments and Implementation
(1) Performance-oriented elite pathway	Talent identification and selection	Identifying and recruiting young individuals with potential for competitive sports development	**Authority** (regulatory selection criteria); **Organisation** (state-run sport schools and training bases)
	Professional coaching team development	Recruitment, training and accreditation of specialist coaches to support elite youth athletes	**Authority** (qualification standards); **Organisation** (hierarchical command through sport bureaux)
	Youth sport competition system construction	Establishment of tiered youth competitions linking school, provincial and national levels	**Organisation** (state-organised events); **Treasure** (event funding)
	Sport–education integration for elite athletes	Provision of academic schooling alongside high-performance training to safeguard athletes’ educational entitlements	**Authority** (dual-track regulations); **Organisation** (cross-sector institutional arrangements)
(2) Population-oriented health enhancement	Health promotion and public information	Dissemination of guidance on physical activity, nutrition and healthy lifestyles targeting young people and families	**Nodality** (information campaigns and public guidance)
	Community sport activities and events	Organisation of grassroots events, summer camps and neighbourhood programmes promoting youth participation	**Nodality** (awareness campaigns); **Organisation** (community-level delivery)
	Public sport facility provision	Construction and upgrading of community sport venues, fitness trails and open public spaces accessible to young people	**Treasure** (capital investment); **Organisation** (municipal delivery)
(3) Education-integrated holistic development	School physical education and health curricula	Embedding of physical education and health-related content within the formal school curriculum, including instructional hours and learning standards	**Authority** (curriculum mandates); **Organisation** (school-based delivery)
	After-school sport provision	Provision of extracurricular sport activities, clubs and supervised play within or beyond school premises	**Organisation** (cross-departmental coordination); **Treasure** (subsidies for after-school services)
	Teacher training and PE workforce development	Pre-service and in-service training of physical education teachers and the expansion of the qualified PE workforce	**Authority** (professional standards); **Treasure** (training funding)
	School sport facilities and resources	Investment in school-based sport infrastructure, equipment and learning resources	**Treasure** (capital and recurrent funding); **Organisation** (education authority delivery)
	Sport–education integration mechanisms	Cross-sectoral institutional arrangements linking the education and sport systems, including joint policy-making, shared standards and inter-agency coordination	Authority (joint regulations); Organisation (inter-departmental coordination platforms)

The coded data were segmented temporally. Following the developmental trajectory of China's political and socio-economic governance — whereby the National Congress of the Chinese Communist Party (CCP) sets sectoral priorities at each historical juncture — three consecutive Party Congresses were adopted as phase boundaries (2012–2016; 2017–2021; 2022–present). Shifts in policy priorities were identified by comparing changes in the frequency distributions and association strengths of the three primary codes, their secondary codes, and the corresponding policy instruments across the three phases. For instance, in the first phase, which was oriented towards Olympic medal acquisition, secondary codes under Primary Code (1) relating to the “development of professional coaching teams” occurred with notably high frequency, whereas secondary codes under Primary Codes (2) and (3) were comparatively sparse. By contrast, in the third phase, secondary codes under Primary Code (3) concerning “school physical education and health curricula” and “after-school sport provision” had risen markedly in frequency. These patterns reflect not only substantive shifts in policy priorities but also a recalibration of governance mechanisms, instrument types and implementation channels over time.

A deductive iterative approach was adopted for the coding process. Initial coding framework was developed on the basis of the existing literature and a preliminary reading of selected policy texts. Two coders applied this framework to independently code a randomly selected sample of 20 policy documents (approximately 19% of the full corpus). Inter-coder reliability was assessed using Cohen's kappa: the *κ* value for the three primary codes was 0.84, while values for the secondary codes and cross-dimensional indicators ranged from 0.79 to 0.87, indicating near-perfect agreement (45). Discrepancies were resolved through structured discussion and consensus, after which the refined framework was applied to the full corpus by the lead coder. At this stage, the second coder conducted periodic spot checks to guard against coding drift and to ensure the reliability and consistency of the results.

## Results and discussion

### Overview of youth sports policy issuances

#### Levels of authority in youth sports policies

This study classified 105 youth sports policies within China's unique legal system, which comprises a four-tier structure of the *Constitution*, *laws*, *State Council regulations*, and *ministry regulations* (46), defining the legislative authority within China's unitary political system [ (47, 48), p. 184]. The first tier is the *Constitution*, which serves as the supreme legal authority, establishing state responsibility for youth physical development through its imperative to ‘cultivate all-round development of youth’ (Art. 46, PRC Constitution) — a non-derogable state obligation that forms the foundational principle for all subsequent policies (49). The second tier comprises statutory *laws* enacted by the National People's Congress (NPC), exemplified by Articles 10 and 24-38 of the Sports Law, which translate constitutional principles into actionable mandates; Article 10's requirement to ‘prioritise youth sports and integrate physical education’ demonstrates how Chinese legislation operationalizes constitutional directives through specific institutional mechanisms, while Chapter 3's provisions reflect legislative intent to systematize youth sports governance (50). The third tier comprises *State Council regulations*, which serve as administrative tools to convert legislative intent into an enforceable policy framework (51). The fourth tier comprises *ministry regulations*, issued by agencies such as the GAS, MOE, and NHC, which provide technical specifications and implementation details for specific administrative areas (52).

**Figure 1:**
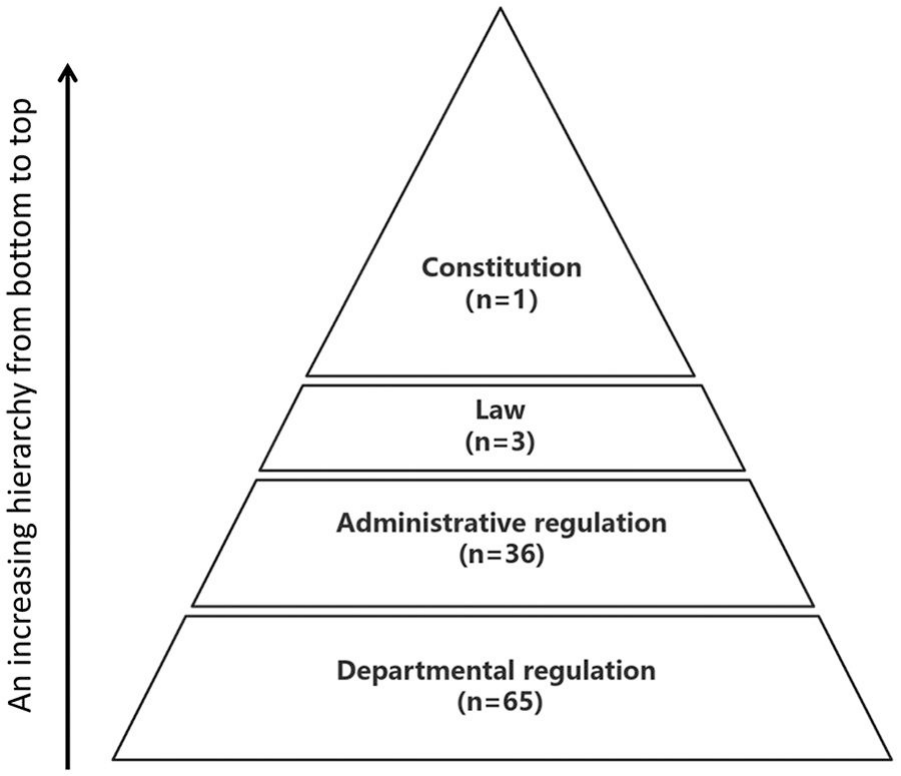
Four-tier legal framework for youth sports policy in China.

It can be observed that China's youth sports policies display a hierarchical progression from the Constitution down to administrative regulations, with legislative density inversely related to temporal durability (Figure 1). The Constitution and statutory laws possess relatively permanent effect, whereas State Council regulations and ministerial regulations (the third and fourth tiers) are characterized by planned obsolescence: their provisions are amended in response to prevailing developmental issues and needs, and they reflect shifts in the CPC's policy priorities. Any amendments to State Council or ministerial regulations must not conflict with higher-level constitutional or statutory law (53). The General Administration of Sport (GAS), the Ministry of Education (MOE), and the National Health Commission (NHC) issue these ministerial regulations as both feedback-driven strategic recalibrations and as compliance conduits between constitutional mandates and administrative implementation. This stratified matrix channels foundational requirements through institutional filtering mechanisms, translating broad obligations into context-sensitive governance practices. Through this process, the framework ensures consistency at the macro level whilst achieving systemic adaptation at the micro level.

Overall, the decade witnessed only one instance of fundamental change at the two higher tiers of constitution and statutory law—the *Sports Law* of the PRC, which for the first time devoted a dedicated chapter to “Youth and School Sport” and thereby established, at the level of national legislation, the supreme legal status of prioritising youth sport development. Beyond this single legislative milestone, however, the policy instruments driving the substantive development of youth sport were almost entirely concentrated at the third and fourth tiers of State Council administrative regulations (*n* = 36) and relevant ministerial regulations (*n* = 65). Together, these two tiers account for 96.2% of the total sample (*n* = 105), and were continuously generated by the State Council and its relevant competent authorities through regulations, ministerial rules, opinions and circulars. This policy structure constitutes a paradigmatic manifestation of the seriality depicted by incrementalism: rather than attempting a one-off reconstruction of the entire youth sport governance system through a single overarching statute, decision-makers have, drawing on the functional remits of individual ministries and addressing specific policy problems as they arise, proceeded through sustained ministerial rule-making to enact continuous marginal adjustments and cumulative revisions to existing institutional arrangements.

It is precisely through this cumulative process of successive limited comparisons (20) that more than one hundred tiered policy documents have been linked and layered upon one another, jointly bringing about these directional substantive shifts in youth sport policy: from a supplementary role subordinate to school education to systemic reconstruction of youth sport; from separation between sport and education to integration of sport and education; and from a single-purpose orientation toward physical robustness to the health-first cultivation of the youth.

#### Regularity of annual issues of youth sports policies

**Figure 2:**
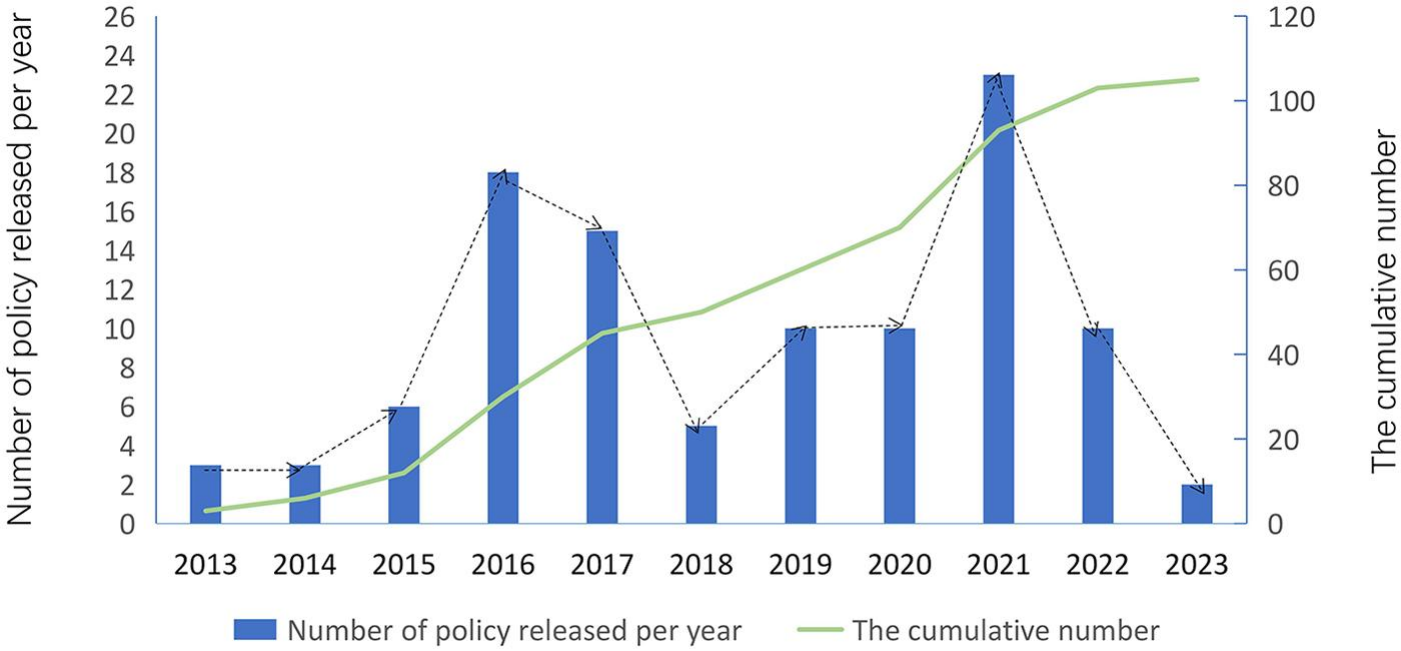
Number of annual youth sport policy releases from 2013 to 2023.

This study analysed the overall development trend of youth sports policies based on the 105 youth sport policy texts. Figure 2 shows the annual and cumulative number of youth sports policies published in China from 2013 to 2023. The data reveal that the annual releases of youth sports policies are characterised by a distinct M-shaped wave pattern, and the cumulative number of releases shows a continuous upward trend. Two notable peaks are observed in the M-shaped wave pattern, occurring in 2016 (*n* = 18) and 2021 (*n* = 23). These surges are intrinsically linked to the launch of China's 13th (2016–2020) and 14th (2021–2025) Five-Year Plan (FYP) cycles—the nation's core strategic planning mechanism since 1953 that establishes phased development priorities across all policy domains (54, 55).

This pattern is consistent with incrementalist expectations: policy activity intensifies at institutionalised review points (Five-Year Plan launches) rather than occurring as abrupt departures from existing frameworks (20). Each FYP cycle served as a policy accelerator, generating predictable surges in policy issuance approximately every five years (55–58). The next anticipated policy peak is likely to emerge following the release of the 15th Five-Year Plan, around 2026.

### Evolutionary phases of youth sports policy and political context

The analysis of the 105 policy texts, combined with the temporal segmentation aligned to Party Congress cycles, reveals three distinct phases in the evolution of youth sport policy priorities. To validate the three-phase partition, the relative frequency of the three primary codes was compared across the three sub-periods. During the 18th NCCPC period (*n* = 19), clauses coded under Primary Code (1), “Performance-oriented elite pathway”, accounted for 58.3% of all coded segments, while Primary Code (2), “Population-oriented health enhancement”, and Primary Code (3), “Education-integrated holistic development”, comprised 23.6% and 18.1% respectively. By the 19th NCCPC period (*n* = 63), this distribution had shifted appreciably to 37.9%, 34.5% and 27.6%. By the 20th NCCPC period (*n* = 23) it had moved further still, to 21.4%, 30.2% and 48.4%, with Primary Code (3) emerging as the dominant category, lending support to the substantive claim that the three NCCPC periods demarcate genuinely distinct policy-priority regimes rather than arbitrary cut-points. Table 2 summarises the key policies and their developmental focus during each phase.

**Table 2:** Key policies for youth sports and its focus and political background 2013–2023.

Phases	Key policies	Issuing body	Main effects	Background
*The 18th NCCPC (2012–2016)*	*Medium-to-Long-Term Plan for Cultivating Reserve Talent in Olympic Competitive Sports (2014)*	the General Administration of Sport	Drawing up a blueprint for reserve talent development across 18 Olympic disciplines	The shrinking of grassroots sports schools and the resulting disruption to the foundation for cultivating reserve talent, which calls for systematic policy design to address the generational gap in talent reserves, to reinforce the talent base for the sustainable development of competitive sports.
	*Opinions on Strengthening School Sports and Promoting the All-round Development of Students’ Physical and Mental Health (2016)*	General Office of the State Council	Systematically enhance the physical fitness and health of all young people	The Decision of the Third Plenary Session of the 18th NCCPC proposed strengthening physical education classes and extracurricular exercises, to promote the physical and mental well-being and fitness levels of youth while providing new vitality for the sustained development of competitive sports.
*The 19th NCCPC (2017–2021)*	*Youth Sports Activity Promotion Plan (2017)*	the General Administration of Sport, the Ministry of Education, the Central Civilisation Office, the National Development and Reform Commission, the Ministry of Civil Affairs, the Ministry of Finance, and the Central Committee of the Communist Youth League	Widely organised sports activities for young people, to meet the growing demand for sporting activities among young people	The shortage of youth sports participation time, lagging development of sports organisations, scarcity of venues and facilities, and insufficient involvement of social forces remain prominent issues. In the report of the 19th NCCPC, President Xi Jinping pointed out the need to widely carry out national fitness activities and further strengthen youth sports and better fulfil the growing demand for sports activities among youths.
	*Guiding Opinions on Strengthening the Cultivation of Reserve Talents for Competitive Sports (2017)*	the General Administration of Sport, the Ministry of Education	Making school sports the basis for the cultivation of reserve talents for competitive sports	The government expected to accelerate the process of building a leading sports nation by promoting the development of competitive sports, which requires a large number of reserve talents and new forces for sustainable development.
	*Opinions on Deepening the Integration of Physical Education and Promoting the Healthy Development of Youth (2020)*	the General Administration of Sport, the Ministry of Education	Make sports and education fully integrated in terms of value, function and purpose, and serve the growth of youth jointly	Physical education has long been on the margin of the school education system, and the function of physical education in nurturing students is absent. Based on the concept of the all-round development of youth's physical and mental being, the government seeks to promote the coordinated development of youth's school learning and physical exercise, and has put forward the concept of the “*ti jiao rong he*”, better integrating physical training into school education.
	*Opinions on Strengthening and Improving School Sport Work in the New Era (2020)*	General Office of CPC Central Committee and the General Office of the State Council	Proposing the reform concept of school physical education by teaching, practising and competing	Following the introduction of the concept of “*ti jiao rong he*”, the Party Central Committee and the State Council have attached more importance to school sports, and have made detailed requirements for school sports work to support the concept of “ti jiao rong he” in action.
	*Opinions on Further Reducing the Homework and Off-Campus Training Burden of Students in Compulsory Education (2021)*	General Office of CPC Central Committee and the General Office of the State Council	Reducing the burden of schoolwork and homework on students, allowing them more time to participate in after-school sports activities	Traditional exam-oriented education brings heavy homework burden and extracurricular cramming classes, students have no time to participate in outdoor activities, and long-term sedentary behaviour leads to a rapid rise in myopia and obesity rates, resulting in serious health problems for young people.
*The 20th NCCPC (2022-current)*	*Opinions on Building a Higher-Level Public Service System for Sports and Fitness (2022)*	General Office of CPC Central Committee, General Office of State Council	Optimising the allocation of public service resources for youth fitness	The report of the 20th NCCPC pointed out the need to establish a comprehensive support system for youth sports development. Building a higher level of public service system for youth fitness is a cornerstone for it.
	*Opinions on Implementing the Student Physical Fitness Enhancement Programme (2025)*	Ministry of Education, National Development and Reform Commission, Ministry of Finance, Ministry of Human Resources and Social Security, General Administration of Sport	Enhance the school sports system to promote students’ healthy growth	Implementing the educational concept of ‘health first’, addressing societal concerns regarding enhancing young people's physical fitness, and resolving practical challenges in school sports such as insufficient activity time, substandard training quality, and shortages of teaching staff and facilities, thereby improving students’ physical and mental wellbeing.

#### The 18th NCCPC 2012-2016: cultivation of competitive sports reserve talent

The development of youth sports in China has always been comprehensive, as has its policy content, which consists of two main segments, one for school sports and one for competitive sports. During the period of the 18th CPC National Congress (2012–2016), the content analysis revealed that policy texts predominantly emphasised the cultivation of competitive sports reserve talent, with school sports serving this objective to a considerable extent. The government expected to accelerate the process of building a leading sports nation by promoting the development of competitive sports, which requires a large number of reserve talents and new forces for sustainable development.

The policy design in this period reflects that the state placed greater emphasis on supporting the realisation of competitive sport goals in youth sport development, which was consistent with the prevailing national strategy at the time (59, 60). Throughout this period, both the formulation and implementation of youth sports policies were driven by a logic: to strengthen China's competitive sports pipeline through systematic talent identification and development (61).

However, while sports education resources were disproportionately allocated towards potential reserve talents, the inclusive physical activity needs of ordinary students were overlooked to a certain extent. Burdened by intense academic pressures, ordinary students often lacked the motivation to engage in sports activities, with physical education curricula in schools relegated to a marginalised status. Consequently, the physical fitness and overall health of the majority of adolescents exhibited increasingly severe concerns. The monitoring data from the GAS in 2014 showed that the obesity rate of 7–18 years old reached 12.5% in urban areas and 8.5% in rural areas, and the myopia rate was more than 70% (62). By 2016, urban and rural obesity rates climbed to 21.03% and 14.23% respectively, while the national poor vision detection rate for ages 13–22 averaged 81.33% (63).

The gradual emergence of these health concerns triggered initial policy responses. The State Council issued the *Opinions on Strengthening School Sports and Promoting the All-round Development of Students’ Physical and Mental Health (2016)*, mandating for the first time that ’students must engage in daily physical activity for ≥ 1 h’. This development illustrates the incrementalist dynamic at work. The accumulation of evidence regarding adolescent health deterioration prompted a marginal expansion of policy attention to encompass health promotion alongside the existing competitive talent cultivation framework, rather than replacing it. This gradual broadening of policy scope laid the groundwork for the more substantial reforms of the subsequent period.

#### The 19th NCCPC 2017-2021: integration of sports and education

During the period from the 19th CPC National Congress to the early stage of the 14th Five-Year Plan (2017–2021), youth sport policy entered a stage of strategic development and adjustment. Among the 105 youth sports policies collected in this study, 63 were released during this period, accounting for nearly 60%, showing a trend of intensive policy release. This surge in policy releases not only reflects the transformation of the government's strategic focus on youth sport, but also demonstrates that the youth sport policy framework was constantly being improved and adjusted.

Towards the end of the previous period, a severe health crisis among adolescents had emerged with escalating concerns. The 19th CPC National Congress in 2017 explicitly positioned youth physical and mental health as foundational to realising the Chinese Dream of national rejuvenation. This strategic pivot prompted a reorientation of governmental priorities, expanding youth sports policy from its previous emphasis toward a dual emphasis on educational development and health promotion.

In November 2017, this shift materialised when the GAS, alongside the MOE and five other departments, jointly issued the Youth Sports Activities Promotion Plan. The policy explicitly prioritised integrating school-based and community sports to expand youth participation, cultivate sustainable exercise habits, and enhance holistic health, moving beyond the narrow objective of elite athlete cultivation.

A critical development during this period was the emergence of the “*Double Reduction” policy* in 2021. Under the traditional exam-oriented education model in China, both parents and students had long been subjected to significant academic pressure (64). Many parents enrolled their children in after-school tutoring programmes, further limiting opportunities for physical activity and outdoor engagement (65, 66). Prolonged sedentary behaviours contributed to persistently high rates of myopia and obesity (67–69). The “*Double Reduction” policy*, by curtailing homework volume and eliminating a substantial number of off-campus tutoring institutions, freed up temporal and spatial resources for students to engage in physical activities (70). Although the policy's full text seldom mentions youth sport directly, it elevated concerns for adolescent physical and mental health to core developmental objectives, thereby creating essential conditions for the reintegration of sport into the education system (71).

Concurrently, the government introduced the concept of sports-education integration (Ti Jiao Rong He) through the 2020 joint policy documents issued by GAS and MOE. Based on the concept of the all-round development of youth's physical and mental wellbeing, this framework sought to promote the coordinated development of youth's school learning and physical exercise (72–74). This represented a further incremental expansion of policy scope, embedding health promotion more deeply within the educational infrastructure.

#### The 20th NCCPC 2022-current: emphasis on ‘health-first’ principle

Entering the phase of the 20th CPC National Congress, the health-first educational principle was positioned as the core guidance for advancing reforms and development in school sports. Within this framework, the sports-education integration was regarded as the pivotal pathway to realising this principle.

The newly revised Sports Law in 2022 addressed the legal vacuum in youth sports at the statutory level (second tier). This revised law amended the original “School Sports” chapter to “Youth and School Sports”, explicitly establishing ‘the state's priority development of youth and school sports.. ensuring adequate physical exercise time for young people and promoting their physical and mental wellbeing’ as statutory objectives. This legislative amendment provided the highest level of legal basis for health-oriented youth sport policies and, together with the policies on sports-education integration, formed the contemporary youth sports policy system.

The content analysis of policies issued during this period confirms the consolidation of health as the primary policy priority. School sports dominated a substantial portion of youth sports policies, and the health-first principle was implemented across the youth sports policy framework. Moreover, the frequency of cross-sectoral collaboration in youth sports governance escalated, with joint issuances by GAS, MOE, the National Health Commission, and other entities comprising 39.1% (*n* = 9) of the 23 policies in this period.

Viewed through the incrementalist lens, the trajectory from the 18th to the 20th NCCPC illustrates a progressive, layered expansion of policy priorities. Competitive talent cultivation was not abandoned but was gradually supplemented and eventually rebalanced by the growing emphasis on health promotion and sports-education integration. Each phase built upon the institutional foundations established in the preceding period, reflecting the characteristic pattern of incremental policy adjustment within China's centralised governance system.

Overall, the findings therefore extend our understanding of youth sport governance particularly in relation to the interplay between political cycles, public health imperatives, and institutional path dependency within a centralised state system. The phased trajectory identified demonstrates that policy reorientation in this domain is neither arbitrary nor abrupt, but is systematically anchored to overarching political periodisation and five-year planning rhythms. In line with existing scholarship on Chinese sport governance, the findings confirm that state-directed policy frameworks continue to serve as the primary mechanism through which youth sport development is steered (59, 61). Theoretically, this study advances the application of incrementalism to non-Western, centralised governance contexts. Existing incrementalist scholarship has been predominantly developed and tested within pluralist, liberal-democratic settings, where policy change is assumed to emerge from negotiation among competing interests (20). By demonstrating that incremental, layered policy expansion operates as a structuring logic within China's top-down governance architecture, this study offers a more precise explanatory pathway for understanding how health promotion became institutionally entrenched in Chinese youth sport governance, rather than remaining a rhetorical aspiration.

### Policy issuers and intersectoral relationships

Youth sports is a highly comprehensive and systematic project. The preceding analysis of policy evolution highlights that China's efforts to develop youth sports are characterised by a remarkable multi-departmental coordination. Under the unified leadership and top-level design of the CPC Central Committee and the State Council, the General Administration of Sport (GAS), the Ministry of Education (MOE), the National Health Commission (NHC), the National Development and Reform Commission (NDRC), and other functional departments are involved in the promotion and implementation of youth sports-related work in different ways.

#### Multi-departmental collaboration in youth sports

**Figure 3:**
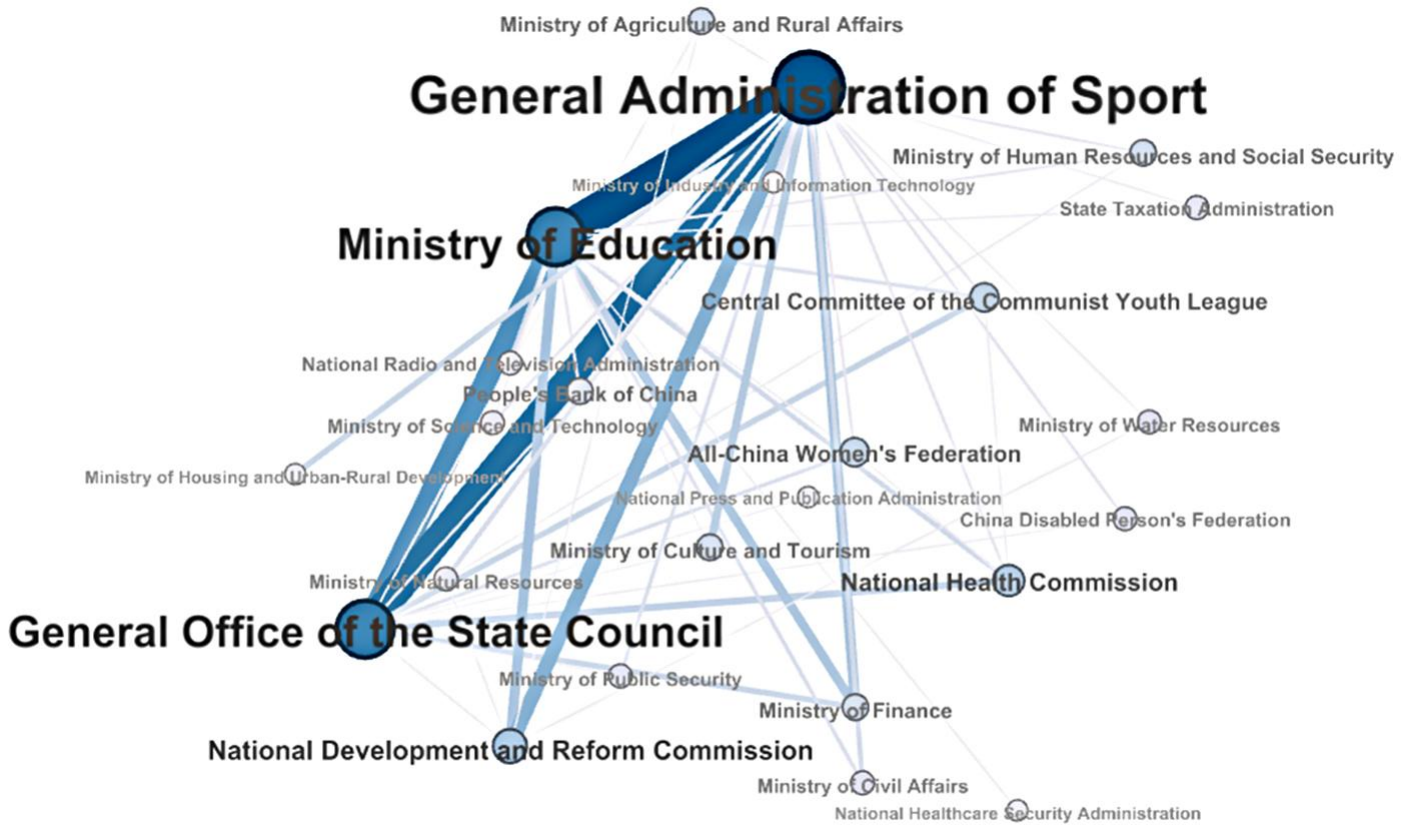
Cooperation network graph between youth sport policy issuers and multiple departments in China.

To examine the relative involvement of different departments in youth sports governance and assess the strength of inter-agency collaboration, this study adopted the social network analysis (SNA) approach with *Gephi 0.10.1* to quantitatively analyse the issuance of youth sports policies by these departments. Figure 3 presents the cooperative network among departments issuing youth sports policies. In this network visualisation, nodes represent policy-issuing departments, and ties between nodes indicate collaborative relationships in policy release. The size of each node corresponds to the number of policies issued by the respective department, while the thickness of the ties reflects the intensity of collaboration between departments.

As illustrated in Figure 3, a wide range of government agencies are involved in youth sports, forming a relatively dense socio-institutional network. A distinct “tripartite structure” can be observed, consisting of the General Administration of Sport of China (GAS), the Ministry of Education (MOE), and the State Council. Internally, this structure operates through a top-down mechanism: the State Council provides strategic guidance and authorization, while GAS and MOE undertake coordinated implementation. The State Council holds a uniquely authoritative position within China's governance framework and plays a decisive strategic role in youth sports development. All other ministries and commissions (including GAS and MOE) operate under its leadership (46, 75, 76).

Four structural metrics confirm and quantify this “tripartite structure”. First, network density (the ratio of observed co-issuance ties to all possible ties among issuing bodies) increased from 0.083 (18th NCCPC period) to 0.211 (19th NCCPC period) and to 0.327 (20th NCCPC period), indicating progressively tighter institutional integration. Second, weighted degree centrality identifies the dominant actors: GAS records the highest weighted degree across all three periods (38, 71, and 46 respectively), but MOE rises sharply from 12 (18th NCCPC period) to 54 (19th NCCPC period) and 41 (20th NCCPC period), overtaking the State Council's General Office as the second-most-active node. Third, betweenness centrality reveals a structural bridging role for the State Council's General Office (normalised score = 0.184 in 20th NCCPC period, the highest in the network), confirming that even when GAS and MOE jointly issue policies, the General Office remains the indispensable strategic intermediary that links sector-specific clusters to non-sport ministries such as Finance, NDRC and NHC. Fourth, modularity Q computed via the Louvain algorithm decreases from 0.612 (18th NCCPC period) to 0.418 (19th NCCPC period) and 0.296 (20th NCCPC period); a falling Q indicates that the previously fragmented clusters — GAS-led competitive sport actors vs. MOE-led school-sport actors — are progressively merging into a cross-sectoral community. Taken together, the rising density, the redistribution of weighted degree, and the falling modularity provide structural evidence for the incremental integration of youth sport governance, complementing the content analytic findings with quantitatively specified network parameters.

The Ministry of Education qualifies as a core actor largely due to the demographic reality that the vast majority of youth in China are formally classified as “students” (77). School sports thus constitute an essential component of youth sports, and the MOE plays a critical role in areas such as Olympic education in primary and secondary schools, physical education curriculum design, and sports facility construction within schools (78–80), thereby securing its position within the core triad.

Externally, this “tripartite structure” functions as a “locomotive” within the broader network of youth sports governance. Other ministries, such as the National Development and Reform Commission (NDRC), the National Health Commission (NHC), and the Ministry of Culture and Tourism (MCT), are coupled to this central engine as “carriages,” interconnected and collectively driven toward the goal of sustainable and healthy development in youth sports.

Notably, the SNA reveals an evolution in collaborative patterns across the three phases. During the 18th NCCPC period, policy issuance was predominantly led by GAS independently, reflecting the competitive talent focus of that era. By the 19th and 20th NCCPC periods, joint issuances between GAS, MOE, and other departments increased substantially, with multi-departmental policies accounting for 39.1% of issuances in the most recent period. This structural shift in the collaboration network mirrors the substantive evolution of policy priorities documented in the content analysis, suggesting that the broadening of policy focus to encompass health promotion has been accompanied by a corresponding diversification of the institutional actors involved in policy formulation.

While multi-departmental collaboration itself is common in youth sports governance across many national contexts, China's model diverges from that of numerous other countries in its overwhelmingly state-led institutional composition. In contrast, many other systems incorporate a wider range of non-governmental actors into their governance frameworks, resulting in broader participation and deeper integration across sectors (81).

#### Challenges and strategies in intersectoral collaboration

The content analysis of the 105 policy documents in this study reveals that a substantial proportion were jointly issued by two or more departments, indicating that intersectoral collaboration is not merely an aspiration but an embedded feature of how youth sports policies are formulated. Yet joint issuance at the formulation stage does not automatically translate into smooth coordination at the implementation stage. A recurring textual feature observed across the corpus is the ambiguity of language regarding which agency is to take the lead, fund, monitor, or be held accountable for specific tasks. When policy texts call for cross-cutting actions without designating a clearly responsible body, the burden of coordination is implicitly transferred to the implementation stage, where departments with similar administrative ranks must negotiate their respective roles *ad hoc*. The 2022 Opinions on Building a Higher-Level Public Service System for Sports and Fitness, for example, calls for establishing a four-tier, cross-age-group and cross-regional youth competition system but does not specify whether the lead responsibility lies with the GAS, the MOE, or another body, which re-enacting the familiar deadlock of “multiple administrations but no one in charge” (82). Comparable difficulties have been documented in the wider literature on collaborative networks, where the absence of a clearly identified coordinating entity is shown to constrain inter-organisational cooperation and resource mobilisation (83, 84), particularly when participating departments share similar administrative levels and power positions (85).

To mitigate the risks that policy ambiguity poses to intersectoral collaboration, future policy drafting could move towards more explicit articulation of inter-departmental responsibilities. At the textual level, this entails specifying, within each jointly issued document, the lead agency, the supporting agencies, and the boundaries of their respective mandates. A clarification step that the existing literature on networked governance regards as a precondition for effective coordination (83, 84). At the institutional level, the State Council, given its overarching position in the policy hierarchy, is well placed to provide top-level design and to mediate goal divergences between the MOE and the GAS, thereby reducing the interpretive load placed on subordinate actors during implementation (86). Comparative experience further suggests that long-term cross-departmental mechanisms are essential for sustaining youth sports development across policy cycles (87). It is important to note, however, that these are forward-looking suggestions inferred from the textual patterns observed in our corpus and from the broader collaboration literature; they are not direct empirical findings of this study, and their effectiveness would need to be examined in dedicated implementation research.

## Conclusion

This study has undertaken a systematic analysis of 105 national-level youth sport policies issued in China between 2013 and 2023, employing bibliometric analysis, content analysis, and social network analysis to trace the evolution of policy priorities across three political cycles. Drawing upon an incrementalist theoretical framework, the study has documented the phased, gradual rebalancing of youth sport policy focus from competitive talent cultivation towards health promotion and sports-education integration.

The findings reveal three distinct evolutionary phases, each aligned with the corresponding Party Congress cycle. During the 18th NCCPC period (2012–2016), youth sport policy was predominantly oriented towards cultivating competitive sports reserve talent, with school sports largely serving as a talent identification mechanism. The 19th NCCPC period (2017–2021) witnessed a strategic expansion, as escalating adolescent health concerns and the introduction of the sports-education integration (Ti Jiao Rong He) concept prompted a dual emphasis on competitive development and health promotion. The “Double Reduction” policy of 2021 further underscored the urgency of addressing youth health through the liberation of time and space for physical activity. By the 20th NCCPC period (2022–present), the health-first principle had been consolidated as the primary policy orientation, codified through the 2022 revision of the Sports Law and reinforced by a significant increase in cross-sectoral policy collaboration.

The social network analysis further demonstrates that this substantive evolution was accompanied by a structural transformation in the policy-making network. The shift from predominantly single-department issuance towards multi-departmental collaboration reflects the broadening of youth sport policy scope and the increasing recognition that health promotion requires coordinated action across education, sport, and health governance systems.

Theoretically, these findings are consistent with incrementalist expectations. Policy changing occurred through a series of gradual, cumulative adjustments at institutionalised review points, principally the Five-Year Plan cycles and Party Congress agendas. Competitive sport talent cultivation was not abandoned but was progressively supplemented and eventually rebalanced by health-oriented priorities. This pattern extends incrementalist theory into the domain of youth sport policy within a centralised governance system, demonstrating that even in contexts of strong state capacity, policy reorientation in youth sport tends to follow incremental rather than radical trajectories.

It is important to note that both competitive sporting achievements and physical and mental health constitute fundamental developmental missions inherent to youth sports. The evolution documented in this study should be understood not as the replacement of one objective by another, but as the gradual rebalancing of priorities in response to evolving societal needs. The selection of elite competitive athletes necessitates a foundation of physically healthy youth, while competitive sports provide positive incentives for youth participation in physical activities. When these two elements mutually reinforce each other to create a virtuous cycle, high-quality and sustainable youth sports development may be genuinely achieved.

This study has several limitations that should be acknowledged. First, the analysis focused exclusively on central-level policy texts and did not examine provincial or local-level implementation, which may diverge significantly from central policy intentions (35). Second, policy texts are not synonymous with outcomes. Given the absence of data on policy implementation, there will inevitably be a gap between the policy documents and the actual outcomes. Third, whilst inter-coder reliability was assessed for the content analysis, the reliance on a deductive coding framework may have constrained the identification of emergent policy themes not anticipated by the existing literature. Fourth, the social network analysis was based on co-issuance relationships and did not capture informal coordination mechanisms or the quality of inter-departmental collaboration.

Future research may address these limitations by incorporating provincial-level policy analysis to examine the degree of alignment between central directives and local implementation. Policy text analysis should be combined with implementation evaluation data to capture not only stated policy priorities but also their actual reach and effects (such as school-level surveys, physical fitness monitoring records, and qualitative fieldwork). Additionally, comparative studies examining youth sport policy evolution across different national governance systems would help to contextualise the Chinese experience within the broader global landscape of youth sport governance.

## Notes

1. The *Sports Law* of the PRC (revised in 2022) was passed and declared on 24 June 2022 and came into force on 1 January 2023 - President of the People's Republic of China Order No. 114.
2. The National Congress of the Communist Party of China (NCCPC) is the most influential political conference in China, which is held every five years and convened by the Central Committee. The meeting's mandate is to discuss and decide on all kinds of important matters for the development of the country – from Constitution of the Communist Party of China (revised in 2022).
3. While constitutional/statutory provisions have perpetual validity (Tiers 1-2), administrative instruments (Tiers 3-4) exhibit temporal dynamism—their issuance frequency and content shifts directly reflect evolving governance priorities in youth sports.
